# *Centella asiatica* extract improves senescence-associated metabolic dysfunction by targeting inflammation in adipose tissue and macrophage in obesity-induced insulin resistance mice

**DOI:** 10.3389/fendo.2025.1589444

**Published:** 2025-07-31

**Authors:** Agian Jeffilano Barinda, Wawaimuli Arozal, Nounik Cheri Dwita, Muhamad Sadam Safutra, Ippei Shimizu, Yung Ting Hsiao, Normalina Sandora, Rani Wardani Hakim, Nurul Gusti Khatimah, Harri Hardi

**Affiliations:** ^1^ Department of Pharmacology and Therapeutics, Faculty of Medicine, Universitas Indonesia, Jakarta, Indonesia; ^2^ Metabolic, Cardiovascular, and Aging Cluster, Indonesia Medical Education and Research Institute (IMERI), Faculty of Medicine, Universitas Indonesia, Jakarta, Indonesia; ^3^ Master Program in Biomedical Sciences, Faculty of Medicine, Universitas Indonesia, Jakarta, Indonesia; ^4^ Department of Cardiovascular Aging, National Cerebral and Cardiovascular Center Research Institute, Osaka, Japan; ^5^ Institute of Medical Education and Research Indonesia, Jakarta, Indonesia; ^6^ Department of Medical Pharmacy, Faculty of Medicine, Universitas Indonesia, Jakarta, Indonesia; ^7^ Drug Development Research Center (DDRC), Indonesian Medical Education and Research Institute, Faculty of Medicine, Universitas Indonesia, Jakarta, Indonesia; ^8^ Doctoral Program in Biomedical Sciences, Faculty of Medicine, Universitas Indonesia, Jakarta, Indonesia; ^9^ Clinical Pharmacology Specialist Study Program, Faculty of Medicine, Universitas Indonesia, Jakarta, Indonesia

**Keywords:** *Centella asiatica*, inflammation, M1 pro-inflammatory macrophage, senescence, white adipose tissue, obesity, insulin resistance

## Abstract

Insulin Resistance (IR) is a complication that frequently occurs in obesity. The inflammation-mediated senescence in White Adipose Tissue (WAT) is important in obesity-induced IR. Centella asiatica (CA) is a potential medicinal plant with anti-aging and anti-obesity properties. Here, we explored the effect of CA on obesity-mediated IR in mice fed with a High Fat-High Fructose (HFHF) diet and treated simultaneously with CA at 150 mg/kgBW (CA150) or 300 mg/kgBW (CA300). The total body mass and visceral WAT weight in both CA groups decreased in comparison with HFHF group alone. We demonstrated that HFHF-diet mice treated with CA300 improved insulin sensitivity and enhanced *Irs-1* activation in WAT. CA300, but not CA150, prevented the senescence phenotype in WAT, represented by decreased Senescence-associated beta-galactosidase (SA-β-Gal) activity and diminished *Cdkn2a* and *Cdkn1a* expression levels at mRNA level. Mechanistically, CA300 prevented the enhancement of *Il6* and *Il1b* mRNA expression levels and macrophage activity in the immunostaining analysis of WAT. *In vitro*, RAW264.7 cells stimulated with high glucose and low dose of Lipopolysaccharides (LPS) also confirmed that CA 200 μg/ml alleviated the expression levels of M1 markers such as *Ccl2*, *Il6*, *Il1b*, and *Tnf* at mRNA level. Our data indicated that CA has therapeutic potential for obesity-mediated IR by suppressing proinflammatory M1 macrophages and preventing inflammation-induced senescence in WAT.

## 1 Introduction

Obesity and its complications significantly contribute to an increase in morbidity and a decrease in quality of life (1). Furthermore, the mortality rate increased by 27% and 93% for grade 1 and 2 obesity, respectively (2). Obesity is a condition of chronic low-grade inflammation that leads to numerous metabolic disorders, such as insulin resistance (3). Various mechanisms contribute to the development of insulin resistance, including increased concentrations of fatty acids, glycerol, hormonal imbalances, and inflammatory cytokines (4).

Obesity, often associated with a high-fat diet, leads to excessive accumulation of triglycerides (TG). TG can build up within adipocytes, resulting in adipocyte hypertrophy and the increased pro-inflammatory cytokines, such as *Il-1b, Il6*, and *Ccl2*. Adipocyte hypertrophy exhibited elevated oxygen consumption, which contributes to adipocyte extracellular matrix remodeling and fibrosis. Altogether, these changes lead to a vicious cycle of adipose tissue dysfunction (5, 6). Adipose tissue dysfunction and insulin resistance are key contributors to cellular senescence. Both conditions can lead to increased reactive oxygen species (ROS) production, activation of the p53/p21 signaling pathway, and elevated *Tnf/Il6* secretion and β-galactosidase activity (7). Specifically, cellular senescence, a cell with a proliferative arrest state, secretes various of pro-inflammatory cytokines, including *Il-1b and Il6*, chemokines, and growth factors to alter tissue microenvironments (8). We previously revealed that senescent endothelial cells induce adipocyte senescence in WAT, reducing *Irs1* expression-mediating systemic insulin resistance through *Il1a*-mediating SASP secretion in EC-specific progeroid mice (9).

*Centella asiatica* (CA), or Indian pennywort, has been traditionally used to increase brain function, lower blood sugar levels, improve blood circulation, and for other uses (10). CA has four main triterpenes (madecassoside, asiaticoside, madecassic acid, and asiatic acid) that exhibit anti-inflammatory, anti-oxidative, and anti-apoptotic properties (11). These effects have been observed in various clinical trials, including in venous hypertension (12), diabetic neuropathy (13), and vascular cognitive impairment (14). Moreover, CA antioxidant properties could prevent aging in human dermal fibroblasts (15). In this study, we explored the effects of CA supplementation on the metabolic phenotypes in obesity-induced insulin-resistant mouse models. We revealed the benefits of dietary CA on this model by alleviating the senescence phenotype.

## 2 Methods and analysis

### 2.1 Diets and CA preparation

Research diets^®^ (D12492) (Rodent Diet with 60% kcal% fat) and D12450J (Rodent Diet With 10 kcal% Fat) from Research Diets, USA were used as a high-fat diet and normal (control) diet, respectively. Diet composition can be seen in **Supplementary Figure 1**. Fructose 25% was made by diluting 25 mg fructose powder (Sweet Food Supply, Indonesia) in 100 mL of drinking water. CA ethanol extract was bought from PT. Plamed (China) and produced following the manufacturer’s procedures. The extraction process of CA began with the crushing of raw plant material into fine powder. Then, the powder was stirred in boiling water and filtered using a plate and frame filter press to separate the solids. The liquid extract was then purified using column chromatography with ethanol as the eluent. The ethanol fraction was concentrated to collect the final extract, which was used for further analysis. CA extract with a concentration of 7.5 mg/mL was made by diluting 22.5 mg of CA ethanol extract into 3 mL of distilled water. The detailed extraction procedure can be found in **Supplementary Figure 2**.

### 2.2 Animal preparations

This study adheres to the principles and standards of animal experiments and has been approved by our institution’s ethics committee with ethical clearance number KET-562/UN2.F1/ETIK/PPM.00.02/2022. Twelve-week DDY strain mice (Biofarma, Indonesia) were housed at a room temperature of 250C and a 12-hour light and dark cycle. The mice were divided into four groups of eight, six of which were used for an *in vivo* study. This study was conducted for 15 weeks which the mice were divided into a control group that was fed with a standard diet and water *ad libitum*; the negative control (HFHF) group was fed with a HFHF diet; treatment group 1 was fed with a HFHF diet and 150 mg/kgBW/day of CA (CA150) via oral gavage; and treatment group 2 was fed with a HFHF diet and 300 mg/kgBW/day of CA (CA300) via oral gavage.150 mg/kgBW/day and 300 mg/kgBW/day were chosen based on a previous reference of CA in D-galactose-induced senescence mice (16).

### 2.3 Body weight and white adipose tissue weight

The weight of each mouse from every group was measured following a 15-week intervention period. At the end of the experiment, the mice were euthanized by anesthesia using intraperitoneal cocktail injection of ketamine at 100 mg/kg and xylazine at 10 mg/kg. Adipose tissues, skeletal muscle tissues, and liver tissues were obtained for analysis. Afterwards, the white adipose tissues located in the inguinal, retroperitoneal, mesenteric, and gonadal regions were individually assessed in terms of their weight.

### 2.4 Insulin tolerance test and glucose tolerance test

At the end of 13^th^ week of the experiment, an intraperitoneal ITT was conducted by administering 5 IU/kgBW human insulin intraperitoneally. At the end of the 14th week of the experiment, a GTT was conducted by administering 5 µL/gBW glucose (3 g of glucose in 10 mL of PBS) following an 8-hour fasting period. In both procedures, blood glucose levels were measured at different interval time points, such as baseline, 15, 30, 60, 90, and 120 minutes after insulin or glucose induction, via cutting off the tip of the tail.

### 2.5 Real-time quantitative reverse transcription polymerase chain reaction analysis

Mice were sacrificed immediately after the GTT test. Adipose tissue was harvested, homogenized by ultra turrax tissue homogenizer (T-25 Janke and Kullkel, UK) and centrifuged by H-103N centrifugator (Kokusan H-103N, Japan). Total RNA was isolated from adipose by the “Direct Zol RNA midiprep Plus with TRI Reagent” kit. cDNA synthesis reaction was performed by ReverTra Ace^®^ qPCR RT Master Mix with gDNA Remover (Toyobo, Japan). All primer sequences can be seen in **Supplementary Table 1**. mRNA relative expressions were examined by RT-qPCR, using *Rplp0* as the housekeeping gene. The mRNA relative levels were calculated using the Livak Method according to our previous research (17).

### 2.6 Histopathology examination

WAT and liver tissues were fixed with 4% paraformaldehyde (PFA) (Wako Pure Chemical) for 24 h prior to dehydration and paraffin embedding, followed by cutting into 3 μm sections for liver and 5 μm sections for WAT. The liver and WAT sections were stained with hematoxylin and eosin (H&E) to evaluate their structural differences, such as adipocyte diameter for WAT and fatty liver pathology for liver. For the fatty liver phenotype, the assessment staging was performed as previously explained (18). Based on a scoring methodology, the histological pathologics were divided into four stages, as follows: 0: no pathological detected; 1: moderate fatty liver (pathological detected limited in one zone only, such as periportal, midzonar, or centrolobular); 2: severe fatty liver (at least two zones are affected); 3: highly severe fatty liver (all zones are affected). In addition, WAT tissues were stained with F4/80 macrophage antibody immunohistochemical staining (Serotec, Raileigh, NC) at a 1:100 dilution to evaluate the crown-like structure. The analysis was performed by calculating F4/80-positive cells using Image J^®^ software in every field of view (19). Leica DM750 digital microscope (Leica) was used to capture images of the liver and WAT sections at 5 random fields in each sample with a total magnification of 100x.

### 2.7 SA-β-Gal staining

Fresh WAT was pre-washed with PBS and incubated in the SA-β-Gal staining solution (Cell Signaling Technology, USA) at 37°C. The WAT was observed every 30 minutes until a cyanish (blue-green) stain appeared. The density of cyan color per area was determined by photographing the five stained WAT in each group to evaluate SA-β-gal activity and analyze cyan color density using Image J^®^ software (9).

### 2.8 RAW264.7 cell and RT-qPCR

RAW264.7 cells, a mouse macrophage cell line (Riken BRC, Japan), were cultured in low glucose-DMEM medium containing 1.0 g/L D-glucose (Sigma, #D6046) or high glucose-DMEM medium containing 4.5 g/L D-glucose (Gibco, #12430-054), both supplemented with 10% FBS and 1% PS (20). We treated the cells with either DMEM medium supplemented with low glucose (5.5 mM) or high glucose (22 mM) for seven days and subsequently stimulated with lipopolysaccharides (LPS) 0.1 μg/mL for 24 hours to induce pro-inflammatory response from macrophages (21). The cells were pretreated with CA (diluted in DMSO <0.01%) at 200 μg/μL 30 minutes before LPS stimulation in both low and high glucose medium (20). Our detailed experimental design for RAW264.7 cells are displayed in **Supplementary Figure 3**. After 24 hours of LPS exposure, the RNA of the cells was extracted, and RT-qPCR was performed. We analyzed the expressions of *Ccl2*, *Il6*, *Il1b*, and *Tnf* as M1-proinflammatory markers in macrophages, and *Il10*, *Cd206*, and *Mgl1* as M2-anti-inflammatory markers (22).

### 2.9 Statistical analysis

The statistical analyses were conducted using SPSS version 26. The data were presented as the mean value ± the standard error of the mean (SEM). The Shapiro-Wilk test was used to assess data normality. For normally distributed data, one-way ANOVA was applied, followed by Tukey’s *post hoc* test. If the data were not normally distributed, the Kruskal-Wallis test was used instead, followed by Dunn’s multiple comparisons test. The ITT and GTT data were analyzed using two-way ANOVA, with Dunnett’s *post hoc* test for multiple comparisons. Statistical significance was denoted as *p<0.05 and **p<0.01. The graphics were generated using GraphPad Prism version 9.5.1.

## 3 Results

### 3.1 *Centella asiatica* supplementation improves the metabolic phenotype in the obese mice model

**Figure 1:**
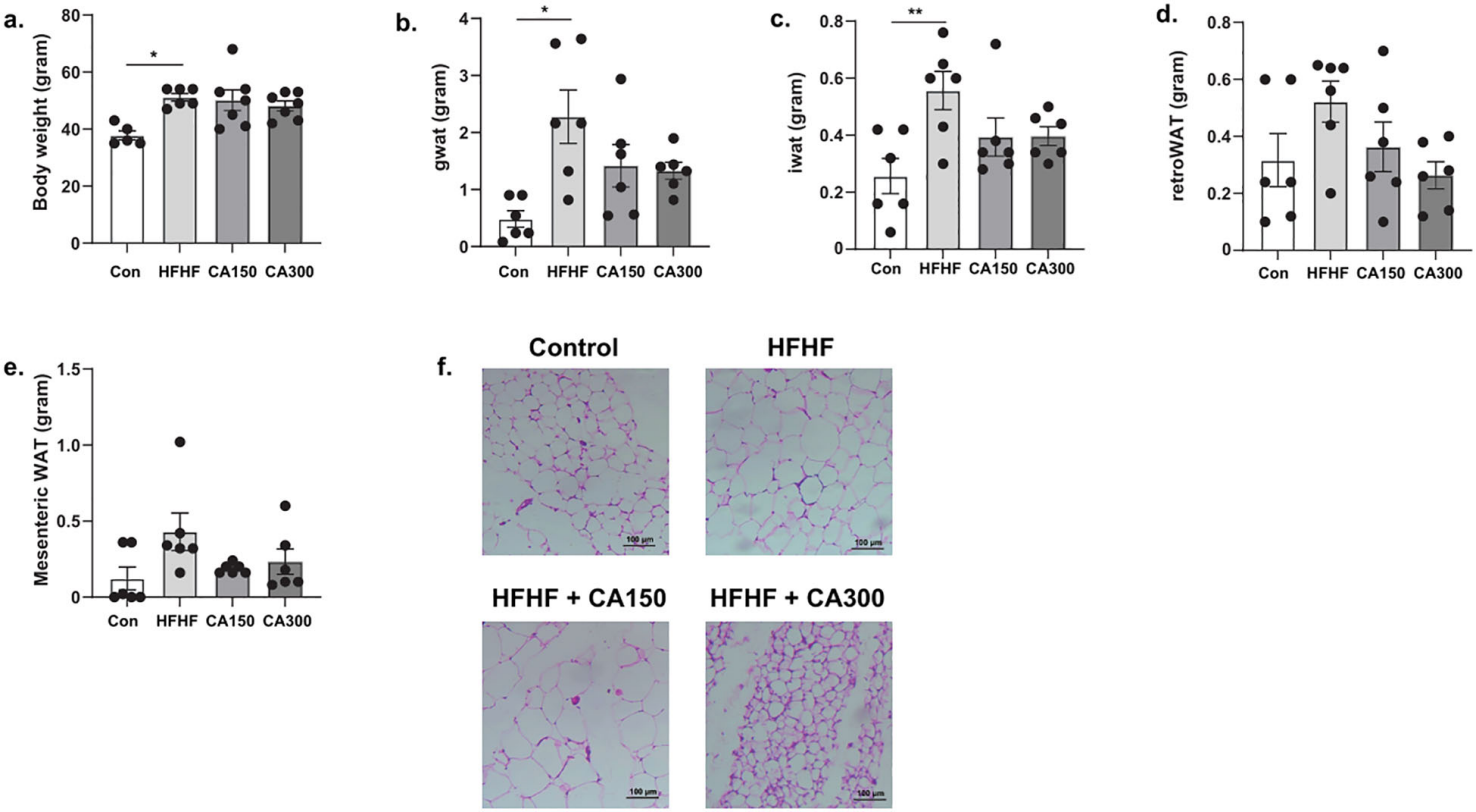
The effects of *Centella* asiatica (CA) supplementation on **(a)** body weight, **(b)** gonadal white adipose tissue (gWAT) weight, **(c)** inguinal white adipose tissue (iWAT) weight, **(d)** retroperitoneal white adipose tissue (retroWAT) weight, **(e)** mesenteric WAT weight, and **(f)** adipocyte diameter based on adipose tissue histopathology analysis at total magnification of 100x (n=5 each group); top left: Con, top right: HFHF, bottom left: HFHF + CA150, bottom right: HFHF + CA300. Con: Control mice, HFHF: High Fat-High Fructose, CA150: 150 mg of *Centella asiatica*, CA300: 300 mg of *Centella asiatica*. *: p<0.05, **: p<0.01.

The mice in the HFHF-diet group exhibited a significant increase in body weight compared to the control group. CA150 and CA300 groups showed a reduction in body weight compared to the HFHF-diet group, although the reduction was not statistically significant (**Figure 1a**). Similarly, the weight of WAT showed a significant increase in the gonadal (**Figure 1b**), inguinal (**Figure 1c**), retroperitoneal (**Figure 1d**), and mesenteric (**Figure 1e**) regions from the HFHF-diet group than the control group, but no statistically significant difference between the HFHF-diet, CA150, and CA300 groups. Histopathological analysis of adipose tissue revealed that adipocyte diameter was larger in mice fed with the HFHF-diet compared to those in the other groups, while smaller adipocyte sizes were observed in the control and CA300 groups (**Figure 1f**).

**Figure 2:**
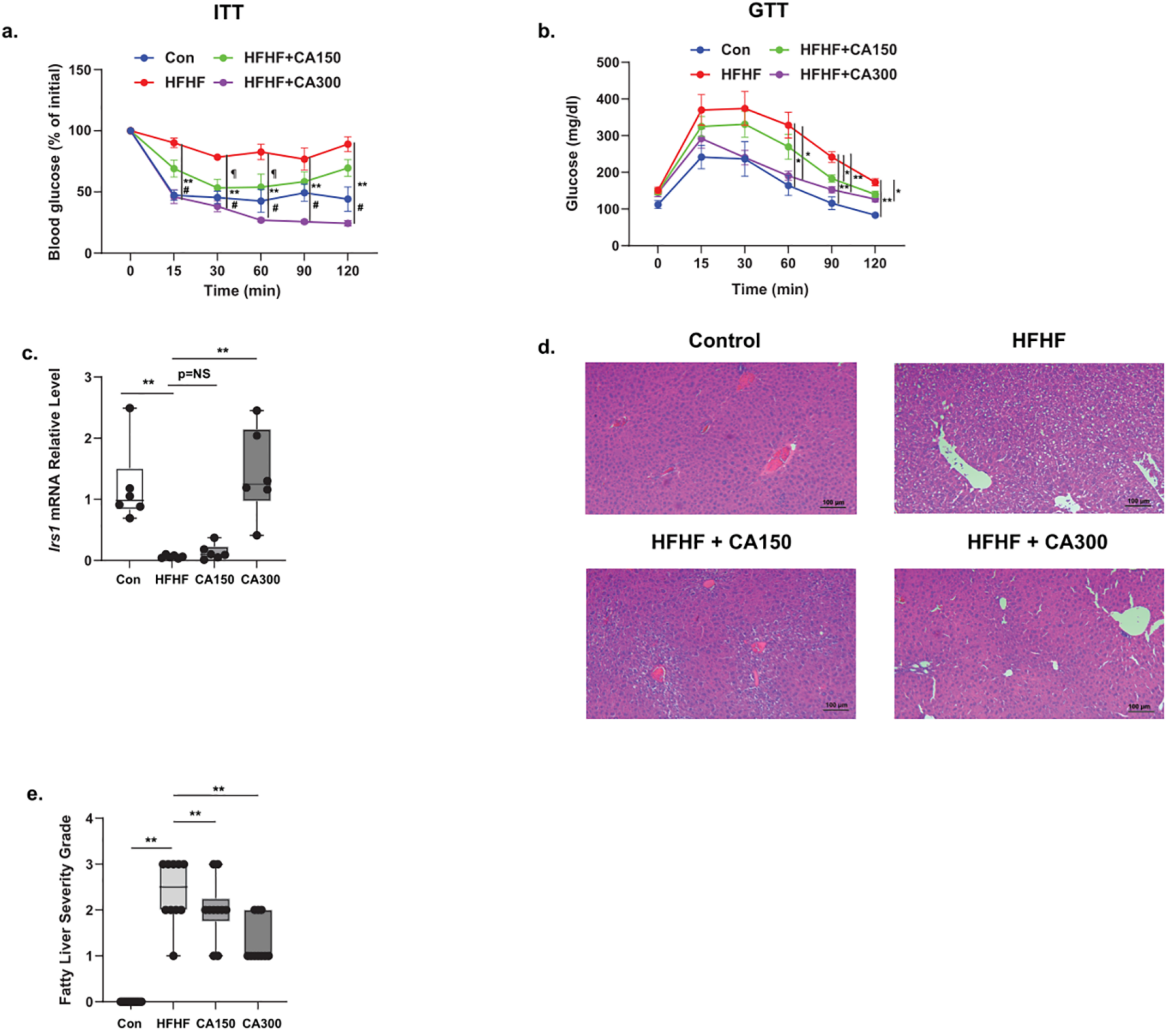
The effect of *Centella* asciatica (CA) supplementation on metabolic properties. **(a)** Intra-peritoneal Insulin Tolerance Test (ITT) and **(b)** Intra-peritoneal Glucose Tolerance Test (GTT). **(c)** IRS-1 mRNA expression level from WAT (n=5 each group). Representative photomicrographs **(d)** and the quantification **(e)** of hematoxylin and eosin (H&E) staining of liver tissues at 100x total magnification (n=5 each group); top left: N, top right: HFHF, bottom left: HFHF + CA150, bottom right: HFHF + CA300. Con: Control mice, HFHF: High Fat-High Fructose, CA150: 150 mg of *Centella asiatica*, CA300: 300 mg of *Centella asiatica*. In **(a, b)** #: p<0.01 between N vs. HFHF groups, **: p<0.01 between HFHF vs. CA300 groups, ¶: p<0.05 between HFHF vs. CA150 groups. In **(c, e, f, h)**, **: p<0.01, NS, Not Significant.

HFHF-diet groups exhibited insulin resistance as indicated by the ITT (**Figure 2a**) and GTT results (**Figure 2b**). Moreover, mice fed with HFHF-diet also showed a reduction in *Irs1* expression in WAT isolated. Of note, CA300, but not CA150 groups, significantly enhanced *Irs1* mRNA expression level (**Figure 2c**). Additionally, both CA150 and CA300 treatments significantly prevented the fatty liver phenotype induced by HFHF based on liver histopathology analysis (**Figures 2d, e**). These data indicate that CA supplementation was able to improve the metabolic impairment in the HFHF-treated mice model.

### 3.2 *Centella asiatica* treatment prevents the senescence phenotype in white adipose tissue isolated from HFHF-diet groups

**Figure 3:**
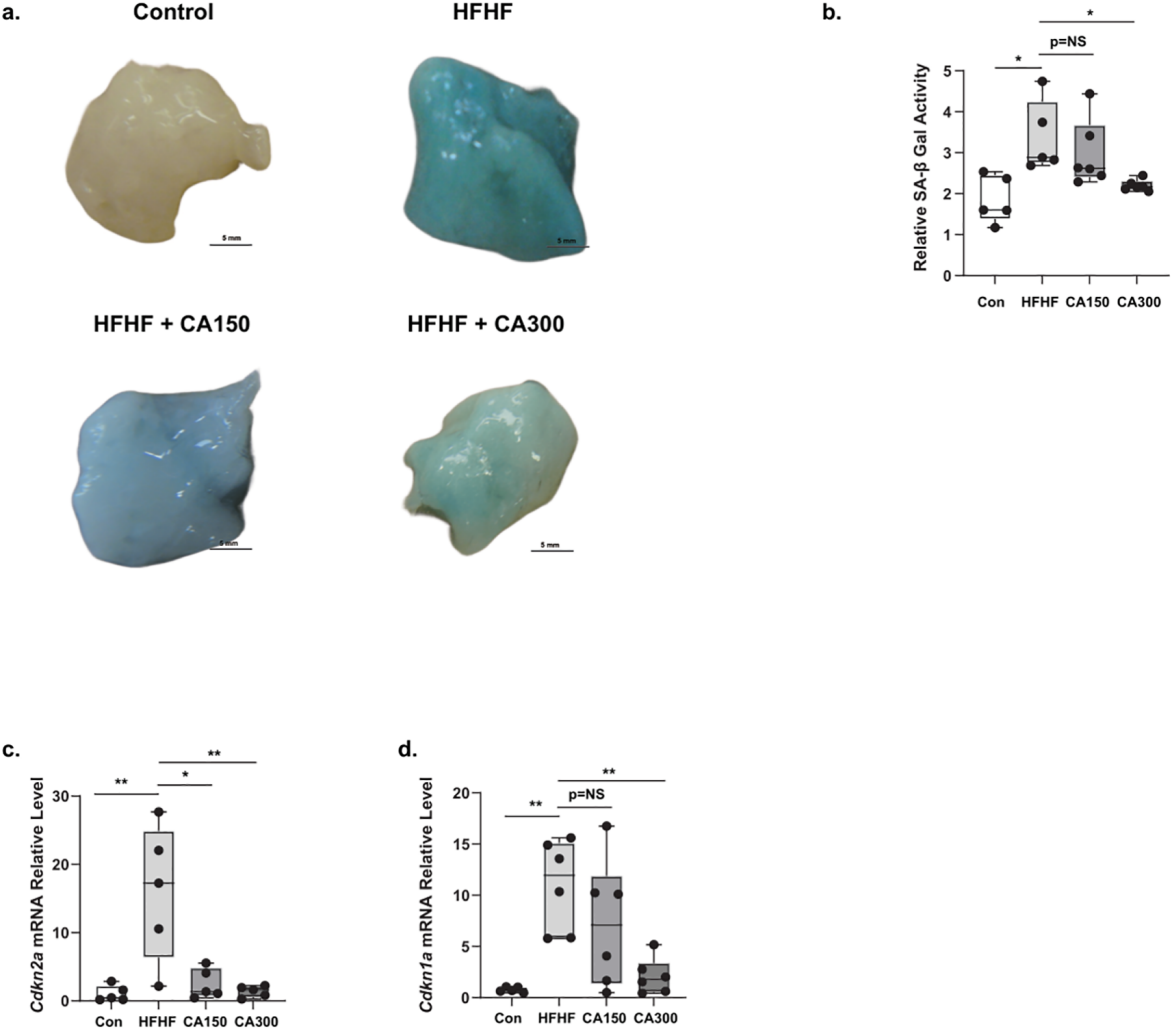
The effect of *Centella asciatica* (CA) on senescence phenotype and factors. SA-β Gal Staining **(a)** and its quantification **(b)** in WAT isolated from normal diet (Control) (top-left), High Fat High Fructose (HFHF) diet (top-right), *Centella asciatica* 150 mg (CA150) + HFHF (bottom-left), and CA 300 mg (CA300) + HFHF (bottom-right) (n=5 each group). **(c)** The effects of CA supplementation on the *Cdkn2a* mRNA expression level, and **(d)** *Cdkn1a* mRNA expression level (n=5 each group). Each bar represents the mean relative mRNA expression ± SEM. Con: Control mice, HFHF: High Fat-High Fructose, CA150: 150 mg of *Centella asiatica*, CA300: 300 mg of *Centella asiatica*. *: p<0.05, **: p<0.01, NS, Not Significant.

We previously showed that senescence phenotype in WAT may induce insulin resistance in mice (9). Based on our findings, SA-β-Gal activity was detected in WAT of the HFHF-diet group. CA300 supplementation significantly suppressed the activity of SA-β-Gal, as indicated by a decrease in the “cyanish” appearance in WAT compared to the HFHF-diet group (**Figures 3a, b**). In the HFHF-diet group, the mRNA expression levels of *Cdkn2a* (**Figure 3c**) and *Cdkn1a* (**Figure 3d**), the Cyclin Dependent Kinase (CDK) inhibitors, were consistently increased, while CA300 treatment significantly reduced the expression of both CDK inhibitors. These data suggest CA treatment may potentially inhibit the senescence progression in WAT following HFHF administration, with CA300 treatment appearing to be potentially more effective.

### 3.3 *Centella asiatica* treatment alleviates inflammation phenotype in white adipose tissue after HFHF administration

**Figure 4:**
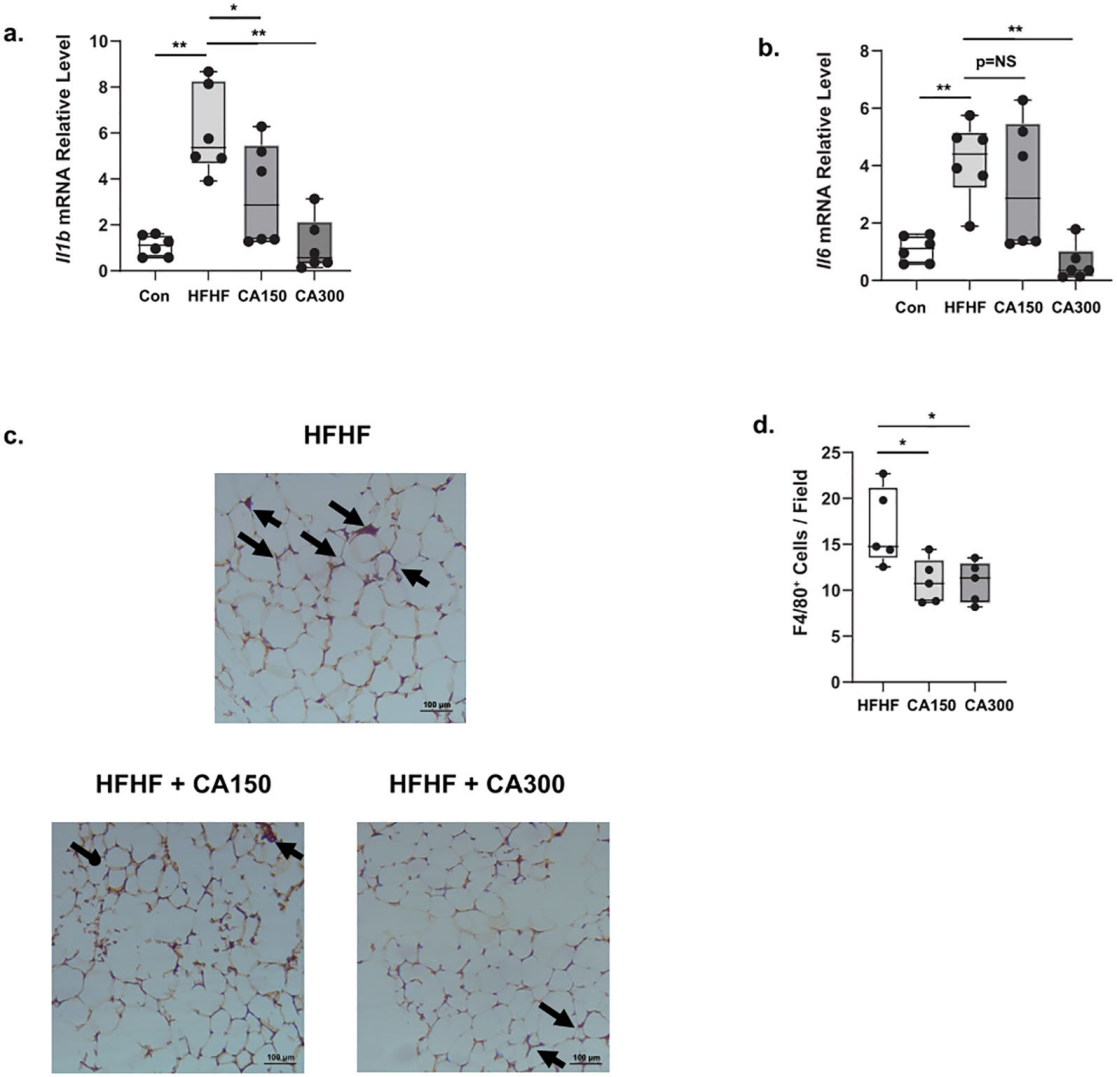
The effect of *Centella asciatica* (CA) on inflammation phenotype in White Adipose Tissue (WAT); n=5 each group. **(a)** The effects of CA supplementation on the *Il1b* mRNA expression level. **(b)** The effects of CA supplementation on the *Il6* mRNA expression level. Representative photomicrographs **(c)** and the quantification **(d)** of F4/80-stained white adipose tissues at 100x total magnification; top: HFHF, Bottom left: HFHF + CA150, bottom right: HFHF + CA300. Con: Control mice, HFHF: High Fat-High Fructose, CA150: 150 mg of *Centella asiatica*, CA300: 300 mg of *Centella asiatica*. *: p<0.05, **: p<0.01, NS, Not Significant.

Pro-inflammatory cytokines, including Interleukin 1 Beta (*Il1b*) and Interleukin 6 (*Il6*), are commonly found in WAT and are characteristic indicators of senescence (9).These cytokines are often associated with the development of insulin resistance in obesity. We observed an increase in the expression of both *Il1b* (**Figure 4a**) and *Il6* (**Figure 4b**) in WAT isolated from the HFHF diet. Both the treatments of CA150 and CA300 significantly decrease the amplification of inflammatory markers following HFHF exposure, but only the CA300 group showed significant downregulation of *Il6* mRNA relative expression levels. Likewise, crown-like structures were observed in WAT isolated from the HFHF-diet group, and both CA 150 and CA300 administration significantly diminished these (**Figures 4c, d**). Collectively, these data strongly suggest that CA treatment suppresses the inflammation phenotype in HFHF-diet mice.

### 3.4 *Centella asiatica* treatment suppresses proinflammatory M1 macrophage polarization *in vitro*

**Figure 5:**
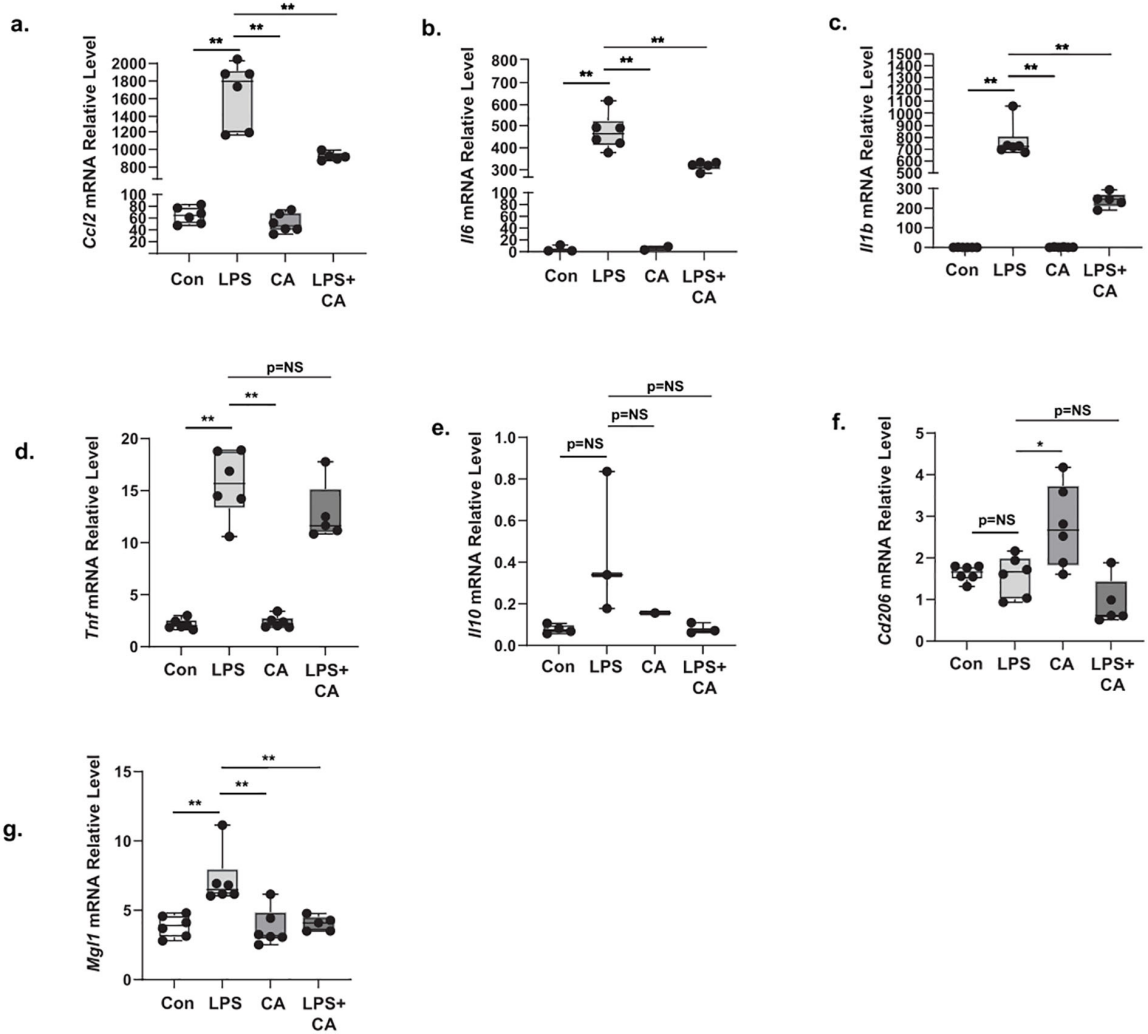
The effect of *Centella asciatica* (CA) on the mRNA expression level of M1 and M2 macrophage markers in RAW264.7 cell stimulated with high glucose and LPS (n=5 each group). **(a)** The effects of CA supplementation on the *Ccl2* mRNA expression level. **(b)** The effects of CA supplementation on the *Il6* mRNA expression level. **(c)** The effects of CA supplementation on the *Il1b* mRNA expression level. **(d)** The effects of CA supplementation on the *Tnf* mRNA expression level. **(e)** The effects of CA supplementation on the *Il10* mRNA expression level. **(f)** The effects of CA supplementation on the *Cd206* mRNA expression level. **(g)** The effects of CA supplementation on the *Mgl1* mRNA expression level. Con: Control cells, LPS: LPS-treated cells, CA: CA-treated cells, LPS+CA: LPS and CA-treated cells. *: p<0.05, **: p<0.01, NS, Not Significant.

To further elucidate the inflammatory molecular mechanism of CA in macrophage polarization, we analyzed both M1-proinflammatory macrophage markers, such as *Ccl2*, *Il6*, *Il1b*, and *Tnf*, and, M2-antiinflammatory macrophage markers, including *Il10, Mgl1*, and *Cd206* in RAW264.7 mouse macrophage cells stimulated with low (5.5 mM) or high glucose (22 mM) in combination with a low dose of LPS (0.1 μg/mL). CA treatment at 200 μg/ml (CA200) only affected the *Il10* mRNA relative expression levels after LPS stimulation under low glucose condition (**Supplementary Figure 4**). However, when RAW cells were exposed with LPS and high glucose condition, CA200 treatment alleviated the mRNA relative expression levels of M1-associated markers such as *Ccl2* (**Figure 5a**), *Il6* (**Figure 5b**), and *Il1b* (**Figure 5c**), but not in *Tnf* (**Figure 5d** **).** In M2 macrophage analysis, the *Il10* (**Figure 5e**) and Mgl1 (**Figure 5f**) mRNA expression levels were comparable between LPS and LPS+CA groups. Additionally, the expression of *Cd206* (**Figure 5g**) showed inconsistency, as it was found to be elevated after CA200 treatment.

## 4 Discussion

In this study, we suggest that CA supplementation may contribute to preventing the pathogenesis of obesity-related metabolic disorders by suppressing senescence-mediated inflammation in WAT. The HFHF-diet mouse model was selected for this study due to its reliability and effectiveness in closely mimicking the metabolic syndromes observed in humans (23). Our model has successfully induced increases in body weight, visceral fat weight, and adipocyte diameter. Moreover, it impaired the insulin sensitivity and downregulated the *Irs1* gene expression in WAT. These results are consistent with previous findings analyzing metabolic parameters in the HFHF-diet mouse model (24, 25). We focused on WAT in our research because obesity is associated with an increased accumulation of senescent adipocytes in WAT (26). These senescent adipocytes may induce senescence-associated secretory phenotype (SASP) to produce a chronic sterile inflammatory microenvironment that results in local and systemic metabolic dysfunction (9). Therefore, pharmaceutical interventions that target senescent cells have been known to prevent organ damage associated with obesity (27).

Previous research demonstrated that CA at doses of 300 and 600 mg/kgBW can reduce body fat accumulation in the HFHF-diet model (28). Previous studies also showed that different doses of CA (50, 100, and 200 mg/kgBW) and asiatic acid (50 mg/kgBW), an active compound found in CA, effectively decreased ITT and GTT in the diabetic mouse model (29, 30). To the best of our knowledge, our study is the first study to investigate ITT and GTT parameters in the HFHF-diet mouse model. We selected CA150 and CA300 because CA300 has demonstrated efficacy in improving metabolic parameters in the HFHF-diet model, while doses below 300 mg/kgBW have proven effective in the diabetic model. We aimed to investigate whether a lower CA dosage would also be effective in the HFHF-diet mouse model. Based on our results, even though CA150 and CA300 treatments failed to reduce body weight, the levels of visceral fat were reduced, and improved insulin and glucose tolerance as indicated by ITT and GTT. At the molecular level, *Irs1* mRNA expression level was significantly upregulated in CA300 groups. Reduced levels of *Irs1* in adipocytes have been identified as a predictor of insulin resistance in human studies (31). CA300 exhibits a greater lowering of the inguinal and retroperitoneal WAT weights, as well as GTT and ITT results. These findings suggest that CA300 has the potential to enhance metabolic properties more effectively than CA150. CA has been known to have a moderate-to-severe hypoglycemic effect, with no published data showing an increase in counter-regulatory hormones, such as glucagon, adrenaline, and cortisol (32). However, those hormones were not analyzed in this manuscript, making it a limitation for this study. We showed the persistent hypoglycemic condition 120 minutes after insulin treatment in the ITT test, particularly in the control (normal-chow diet) and CA300 groups, respectively. This phenomenon was probably due to the relatively high insulin dose (5 IU/kgBW) that we administered to the mice, since we assumed this is a suitable dose in the HFHF model for inducing a decrease of at least 50% from baseline blood glucose levels. We used a 1 IU/kgBW insulin dose in normal-feeding diet mice for the ITT test in our previous study (9). Even this higher insulin dose could achieve that condition; some groups, including the group with *Centella asiatica*, had a persistent hypoglycemic effect.

Previous research showed that CA significantly decreased the expression of the *Cdkn2a* and *Cdkn1a* genes in human dermal fibroblasts and human hepatoma cells (33, 34). To the best of our knowledge, there is no previous *in vivo* study related to this topic. Our study showed that the expression of *Cdkn2a* and *Cdkn1a* in WAT was reduced by the administration of CA300. The upregulated of these gene expression accelerates the senescence process by affecting these core aging pathways, particularly p16^INK4A^/pRB, and p53/p21^WAF1/CIP1^ pathways (35). Consistent with our findings, a previous study demonstrated that asiaticoside, a key active compound of CA, inhibited *Cdkn1a* expression in UV-induced HaCat cells (36). Our SA-β-gal test conducted in WAT showed a “cyan-color” appearance in all the HFHF-diet group, including those treated with CA. Although the CA150 and CA300 groups showed a lesser cyanish color compared with the HFHF-diet group alone. This finding showed that CA can inhibit the increase of lysosomal activity, indicating the inhibition of the senescence process. Another previous *in vitro* study also showed a similar effect in human dermal fibroblasts (37).

Senescence cells disrupt metabolic states and lipid metabolism, leading to metabolic disease (38). Metabolic derangement has two important hallmarks, *Il1b* and *Il6*. *Il1b* is associated with the initiation and progression of obesity-induced insulin resistance (39), whereas *Il6* plays a role in maintaining energy and metabolic balance through its involvement in energy metabolism (40). Therefore, we used these two biomarkers to evaluate the CA ability to prevent the inflammation caused by senescence. Our study demonstrated that the mRNA relative expression levels of *Il1b* and *Il6* were reduced in the CA administration group compared to the HFHF-diet group, confirming the effect of CA in this pathway. This finding aligns with other studies conducted on mouse macrophages and a psoriasis-like skin inflammation mouse model (41, 42).

Our study also discovered that CA supplementation reduced adipocyte diameter on the histopathological analysis. One proven hypothesis is that CA can regulate lipid metabolism-related genes, such as peroxisome proliferator-activated receptor gamma (PPARγ), fatty acid synthase (FAS), cluster of differentiation 36 (CD36), 3- 3-hydroxyl-3-methylglutaryl CoA reductase (HMGCR), and stearoyl CoA desaturase 1 (SCD1) in the HFHF-diet model (28). We proposed an additional mechanism by which CA supplementation reduces adipocyte diameter by regulating *Il1b* and *Il6*, as these interleukins are involved in fat-liver crosstalk and adipogenesis, respectively (43, 44). Crown-like structure (CLS) is a characteristic feature of inflamed fat cells that is a persistent location for macrophage activation (45). The CLS macrophage activation mechanism is believed to occur through elevated levels of MAC-2, also known as galectin-3, a lectin that facilitates macrophage phagocytic and inflammatory responses (45, 46).

We found that CA decreased the number of CLS in WAT. Adipocyte hypertrophy can trigger low-grade inflammation, and CLS numbers are related to increased expression of M1 macrophages (47, 48). Under obesity-related metabolic pathological conditions, macrophages undergo a phenotypic shift towards the M1 pro-inflammatory macrophages. The M1 polarization will induce the secretion of pro-inflammatory cytokines, such as *Il6*, *Il1b*, *Tnf*, and *Ccl2* (48). Therefore, we conducted an *in vitro* study on LPS-induced RAW264.7 cells to gain better insight into the mechanism of CA in macrophage inflammatory responses. CA reduced the expression of M1 macrophage markers, such as *Ccl2*, *Il6*, *Il1b*, and *Tnf*, in high glucose environments. Our results suggest that CA could suppress M1 macrophages. Another study showed that CA can decrease the expression of proinflammatory macrophages is related to the suppression of IL-1 receptor-associated kinase and transforming growth factor-β-activated kinase 1 (IRAK1-TAK1) pathways (42). This finding supports the hypothesis that one potential mechanism by which CA can reduce senescence markers and metabolic phenotypes is through its anti-inflammatory properties, particularly by modulating macrophage activity.

We discovered interesting characteristics in the *IL-10 response in RAW264.7 cells, whereby IL-10* expression was upregulated following LPS stimulation under low glucose conditions but did not exhibit a substantial increase under high glucose conditions. In contrast, *Cd206*, another M2 macrophage marker, was downregulated after LPS induction. The increase in *Il10* expression is consistent with findings from a previous study on the THP-1 macrophage cell line, which also reported elevated *Il10* expression level following LPS stimulation. Additionally, the previous study also found that Cd206 expression was decreased after 24 hours of LPS exposure (49).

## 5 Conclusion

*Centella asiatica* shows anti-senescence effect in obesity-induced insulin resistance (IR) by preventing senescence induction in WAT of an obesity rodent model. This phenomenon effectively improves systemic insulin sensitivity and further suppresses M1-proinflammatory macrophage-induced inflammation. These findings highlight the role of *Centella asiatica* as a promising medicinal compound treatment for obesity-related metabolic disorders.

## Data Availability

The raw data supporting the conclusions of this article are available from the corresponding author upon reasonable request.

## Supplementary material

The Supplemental Material for this article can be found online at: https://www.frontiersin.org/articles/10.3389/fendo.2025.1589444/full#supplementary-material
