# Supplementary figures and images for: Centella asiatica extract improves senescence-associated metabolic dysfunction by targeting inflammation in adipose tissue and macrophage in obesity-induced insulin resistance mice
Source: Front Endocrinol (Lausanne). 2025 Jul 31;16:1589444. doi: 10.3389/fendo.2025.1589444 (PMC12350134; doi:10.3389/fendo.2025.1589444)

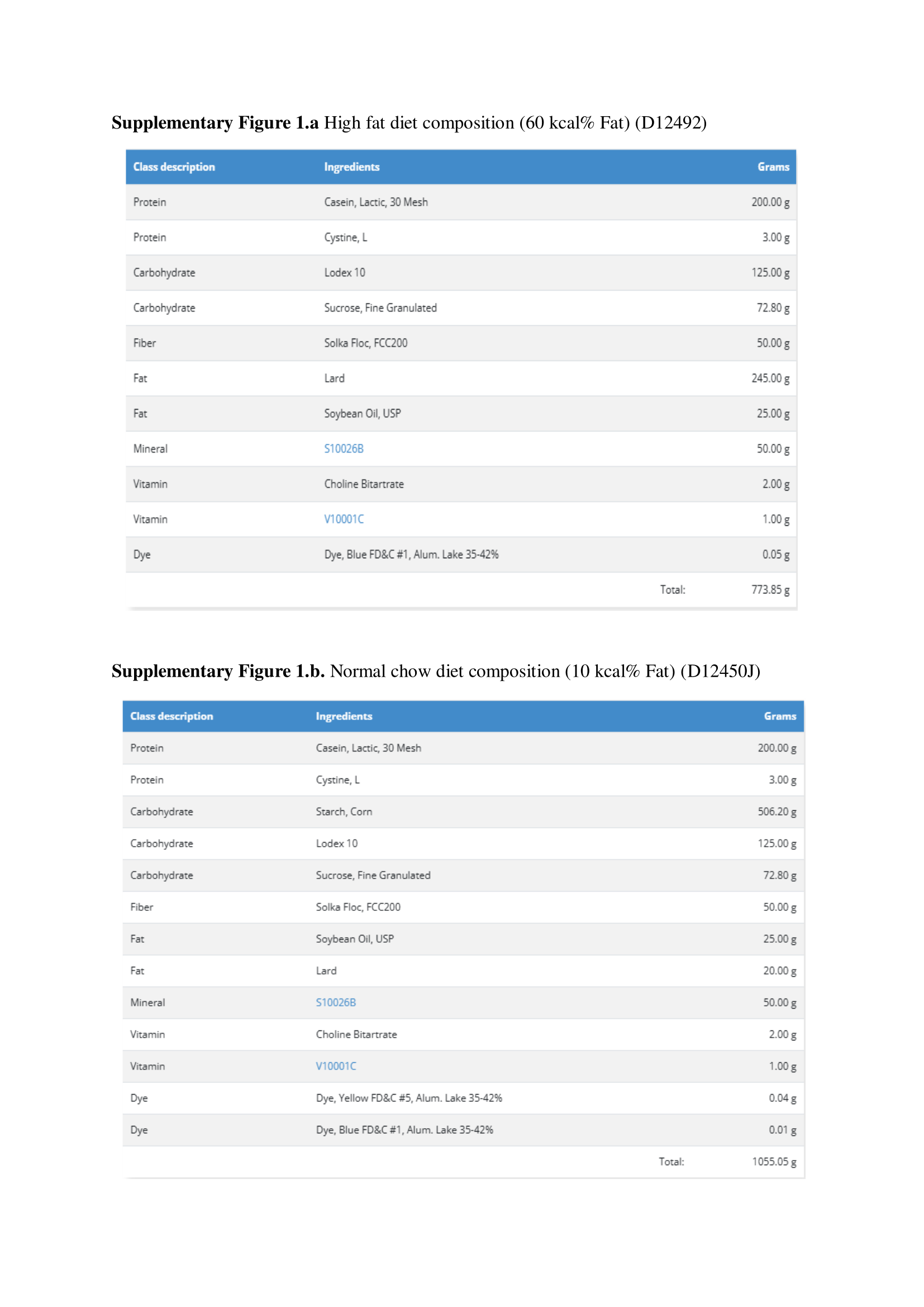

Supplement: Supplementary file 1 [file Image1.tiff]

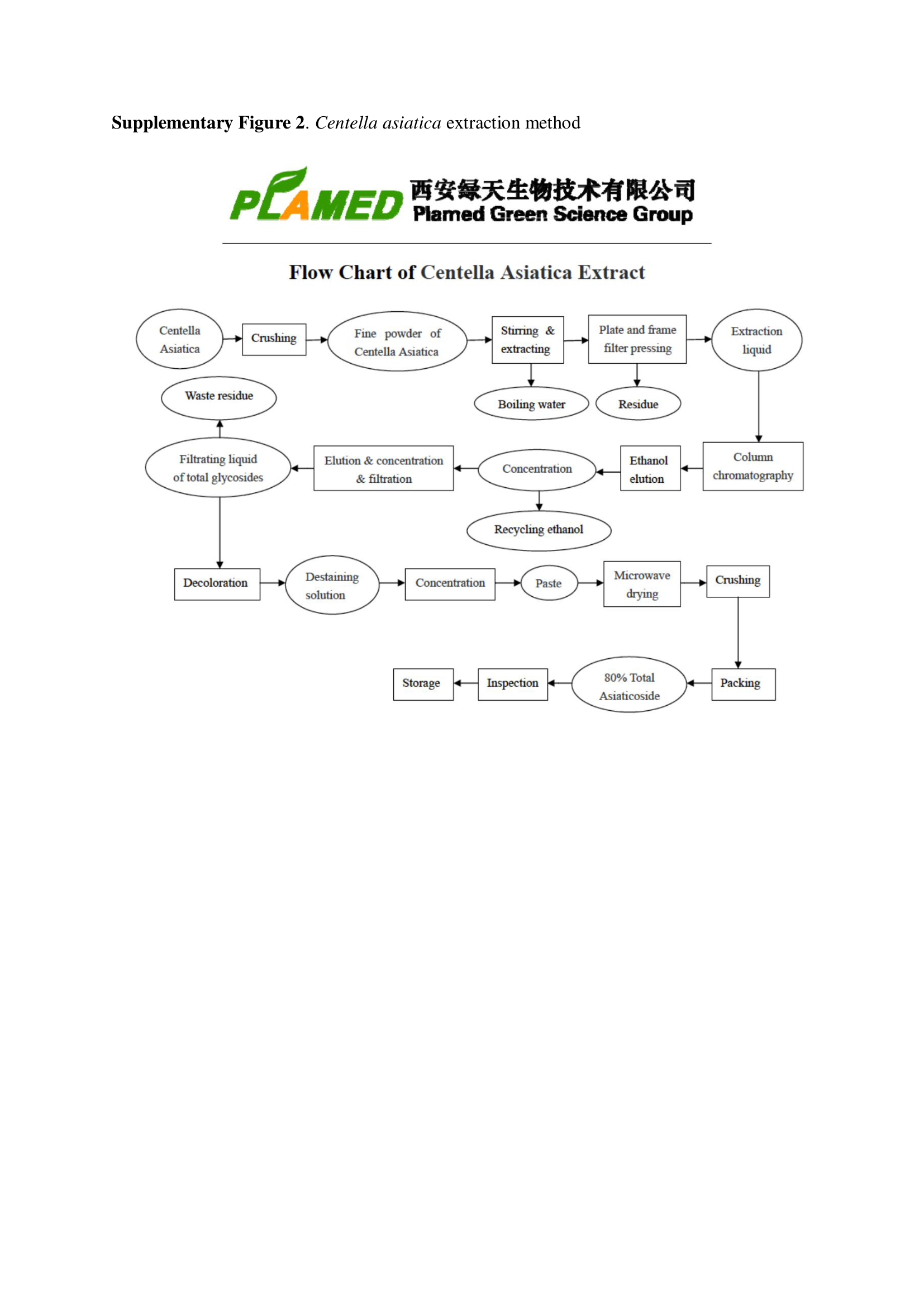

Supplement: Supplementary file 2 [file Image2.tiff]

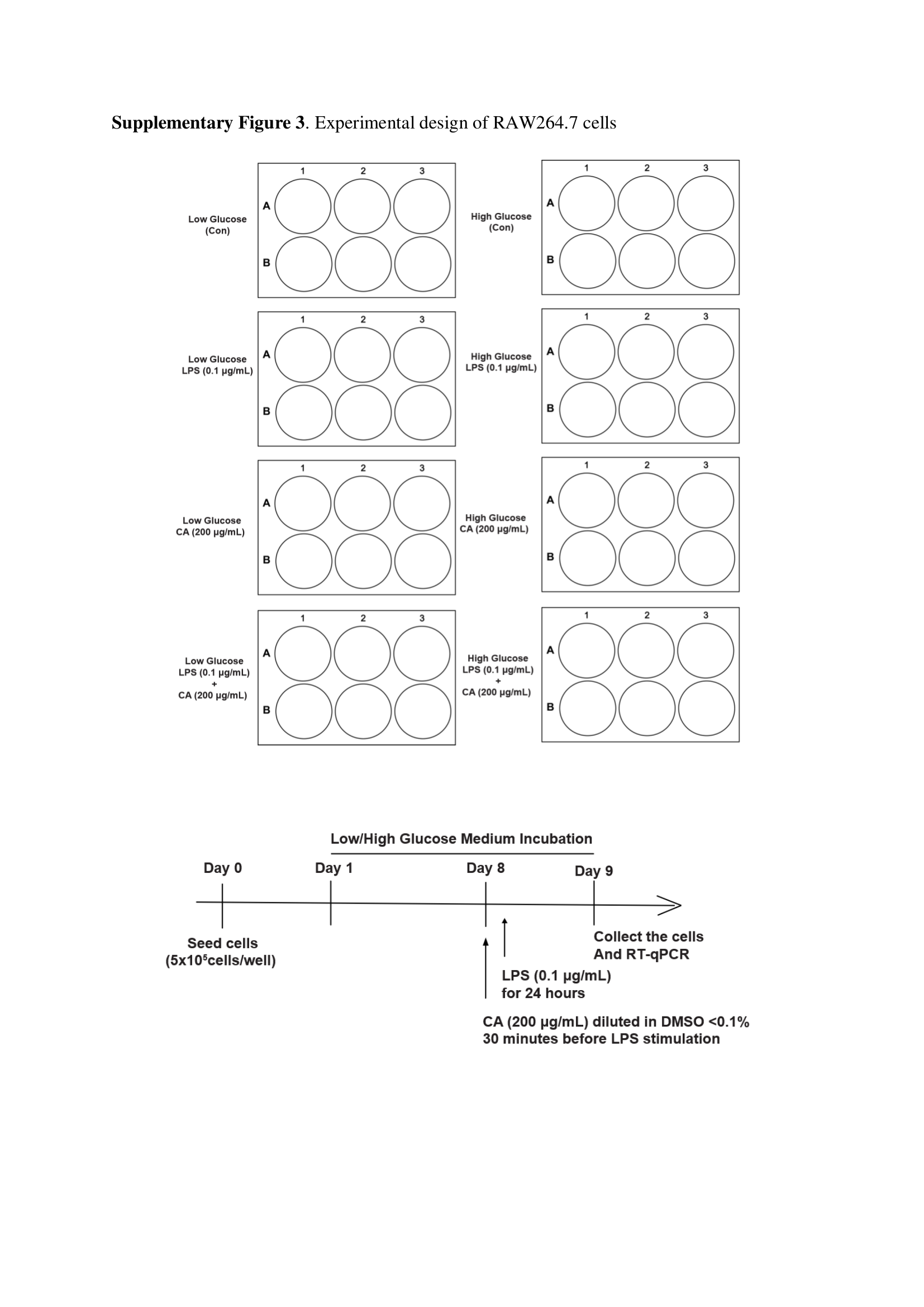

Supplement: Supplementary file 3 [file Image3.tiff]

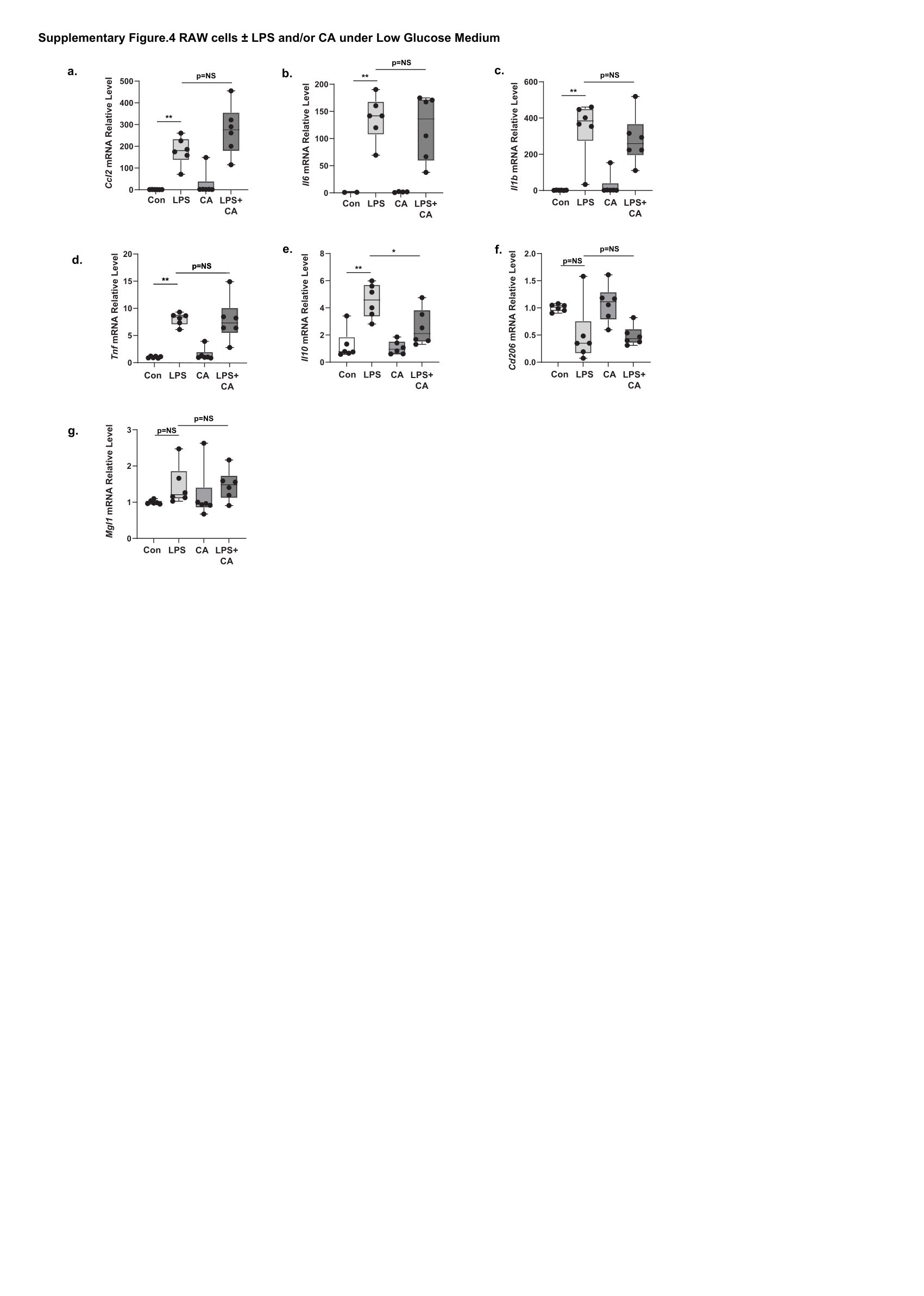

Supplement: Supplementary file 4 [file Image4.tiff]
