# Supplementary material for: Centella asiatica extract improves senescence-associated metabolic dysfunction by targeting inflammation in adipose tissue and macrophage in obesity-induced insulin resistance mice
Source: Front Endocrinol (Lausanne). 2025 Jul 31;16:1589444. doi: 10.3389/fendo.2025.1589444 (PMC12350134; doi:10.3389/fendo.2025.1589444)
Supplement: Supplementary file 5 [file Table1.docx]

**Supplementary Table 1**. Mouse primer sequences for *in vivo* and *in vitro* RT-qPCR

| Gene | Primer | Sequence |
| --- | --- | --- |
| *RPLP0* | Forward | 5’-GATGCCCAGGGAAGACAG-3’ |
|  | Reverse | 5’-ACAATGAAGCATTTTGGATAA-3’ |
| *Irs1* | Forward | 5’-GACGCTCCAGTGAGGATTTAAG-3’ |
|  | Reverse | 5’-GGATTTGCTGAGGTCATTTAGG-3’ |
| *Cdkn2a* | Forward | 5’-GACGGGCATAGCTTCAGCTCAAGCA-3’ |
|  | Reverse | 5’-GCCACATGCTAGACACGCTAGCATCGC-3’ |
| *Cdkn1a* | Forward | 5’-GCCACAGGCACCATGTCCAATCCTGG-3’ |
|  | Reverse | 5’-GCATCGCAATCACGGCGCAACTGCTC-3’ |
| *Ccl2* | Forward | 5’-CATCCACGTGTTGGCTCA-3’ |
|  | Reverse | 5’-GATCATCTTGCTGGTGAATGAGT-3’ |
| *Il1b* | Forward | 5’- GGGGCGTCCTTCATATGTGT -3’ |
|  | Reverse | 5’-ATACAACGGCTCCTCCGTTC-3’ |
| *Il6* | Forward | 5’-GCTACCAAACTGGATATAATCAGGA-3’ |
|  | Reverse | 5’-CCAGGTAGCTATGGTACTCCAGAA-3’ |
| *Il10* | Forward | 5’-CAGAGCCACATGCTCCTAGA-3’ |
|  | Reverse | 5’-TGTCCAGCTGGTCCTTTGTT-3’ |
| *Cd206* | Forward | 5’-CCACAGCATTGAGGAGTTTG-3’ |
|  | Reverse | 5’-ACAGCTCATCATTTGGCTCA-3’ |
| *Mgl1* | Forward | 5’-GAAAACCCAAGAGCCTGGTA-3’ |
|  | Reverse | 5’-AGGTGGGTCCAAGAGAGGAT-3’ |
| *Tnf* | Forward | 5’-TCTTCTCATTCCTGCTTGTGG-3’ |
|  | Reverse | 5’-GGTCTGGGCCATAGAACTGA-3’ |
